# Application of Independent Component Analysis and Nelder–Mead Particle Swarm Optimization Algorithm in Non-Contact Blood Pressure Estimation

**DOI:** 10.3390/s24113544

**Published:** 2024-05-30

**Authors:** Te-Jen Su, Wei-Hong Lin, Qian-Yi Zhuang, Ya-Chung Hung, Wen-Rong Yang, Bo-Jun He, Shih-Ming Wang

**Affiliations:** 1 Department of Electronic Engineering, National Kaohsiung University of Sciences and Technology, Kaohsiung 80782, Taiwan; sutj@nkust.edu.tw (T.-J.S.); i110152102@nkust.edu.tw (W.-H.L.); j111252102@nkust.edu.tw (Q.-Y.Z.); aaa13y@gmail.com (Y.-C.H.); i110152105@nkust.edu.tw (W.-R.Y.); paul56565600@gmail.com (B.-J.H.); 2 Department of Computer Science and Information Engineering, Cheng Shiu University, Kaohsiung 833, Taiwan

**Keywords:** non-contact blood pressure estimation, independent component analysis, Nelder–Mead simplex method, particle swarm optimization algorithm

## Abstract

In recent years, hypertension has become one of the leading causes of illness and death worldwide. Changes in lifestyle among the population have led to an increasing prevalence of hypertension. This study proposes a non-contact blood pressure estimation method that allows patients to conveniently monitor their blood pressure values. By utilizing a webcam to track facial features and the region of interest (ROI) for obtaining forehead images, independent component analysis (ICA) is employed to eliminate artifact signals. Subsequently, physiological parameters are calculated using the principle of optical wave reflection. The Nelder–Mead (NM) simplex method is combined with the particle swarm optimization (PSO) algorithm to optimize the empirical parameters, thus enhancing computational efficiency and accurately determining the optimal solution for blood pressure estimation. The influences of light intensity and camera distance on the experimental results are also discussed. Furthermore, the measurement time is only 10 s. The superior accuracy and efficiency of the proposed methodology are demonstrated by comparing them with those in other published literature.

## 1. Introduction

Advancements in medical and semiconductor technology have facilitated the development of wearable devices that are capable of capturing physiological signals generated by the human body. Electrocardiography (ECG) and photoplethysmography (PPG) are commonly used signals for estimating blood pressure. Kato Y. [1] proposed a non-contact blood pressure measurement model that involves the entire facial vascular structure being viewed as a circuit, indicating that blood pressure-related facial features can be obtained through CEOF analysis. Baker S. [2] proposed a hybrid neural network for accurately estimating blood pressure using only non-invasive ECG and PPG waveforms as inputs. The proposed hybrid neural network combines the feature detection capabilities of temporal convolutional layers and the strong performance on sequential data provided by long short-term memory (LSTM) layers. Ripoll and Vellido [3] proposed a data-driven approach based on the restricted Boltzmann machine (RBM) by utilizing PPG and ECG signals and employing a foot-to-foot algorithm [4] for pulse transit time calculation. However, the model has some drawbacks, such as reduced performance when calibration stops, thus making it unsuitable for clinical settings. Wang et al. [5] proposed a method for blood pressure estimation based on the Multitaper Method (MTM) to extract features from PPG signals. Chen et al. [6] proposed systolic blood pressure (SBP) and diastolic blood pressure (DBP) estimation models based on pulse transit time (PTT) and PPG waveform features. They investigated the influence of each feature and fine tuned the model parameters using a genetic algorithm. The aforementioned researchers used non-contact methods to obtain PPG and ECG signals for different analyses. Fang [7] utilized chrominance signal processing and orthogonal skin-plane methods to reduce noise and to extract image-based photoplethysmography signals. K-nearest neighbor (KNN) and linear regression models were employed to model a blood pressure database based on the frequency phase difference features for full-face-image-based blood pressure measurements. Rong and Li [8] provided a machine learning method that supports vector regression (SVR) to detect BP using iPPG signals. Also, Li et al. [9] introduced an attention-based LSTM machine learning technology that uses iPPG signals. However, some of the published papers mentioned above did not discuss the measurement of distance and time or more complete evaluation metrics, e.g., mean absolute percentage error (MAPE), root mean square error (RSME), promotion of improved accuracy, etc.

This study aims to develop a non-contact blood pressure measurement system by extracting the region of interest (ROI) for obtaining remote photoplethysmography (RPPG) signals. Independent component analysis (ICA) is employed to eliminate artifact signals. Subsequently, physiological parameters are calculated using the principle of optical wave reflection. The Nelder–Mead (NM) simplex method is combined with the particle swarm optimization (PSO) algorithm to optimize the empirical parameters based on body mass index (BMI). Utilizing the direct search strategy of the NM algorithm to finely adjust the particle positions in PSO helps to prevent premature convergence and enhances the likelihood of discovering a global optimal solution. The NM algorithm is more robust than a local search. After PSO finds a promising area, NM can be employed to determine the optimal solution more precisely. Combining the advantages of both methods makes the new NM-PSO algorithm more stable and effective when dealing with multi-peak, high-dimensional, and noisy optimization problems. In summary, the NM-PSO algorithm can effectively address various shortcomings when used independently, particularly in complex optimization problems, by integrating the local search accuracy of NM with the global search capability of PSO.

The structure of this paper is as follows: Section 2 details the research methods and systems description, and Section 3 shows the experimental results. In Section 4 and Section 5, the experimental results are discussed and this study’s main findings and contributions are summarized, respectively.

## 2. Methodologies and System Description

This study aims to enhance computational speed and accuracy by utilizing a webcam for face detection. It captures the pulse wave reflected from the forehead as the ROI and calculates blood pressure values based on the extracted light wave. The methods and experimental procedures that were employed are detailed below.

### 2.1. Face Detection and Recognition

#### 2.1.1. Face Detection and Region of Interest (ROI)

Face detection is a subset of object detection that specifically identifies the presence and location of human faces within an image. It typically involves marking the position of faces with rectangular bounding boxes and may also identify finer facial features such as mouths, eyes, and foreheads.

ROI delineates specific areas from processed images in various shapes, including irregular polygons, circles, ellipses, and rectangles. Multiple operations and functions identify and process these regions, with ROI applications extending to image segmentation, face recognition, and heatmap generation. In this study, face detection is used to extract from the facial region for analysis by following the approach detailed in reference [10]. This method explicitly selects the cheeks and forehead as inputs because these areas are optimal for detecting blood pulse signals. Using the entire facial region as a model input might introduce interference from non-skin elements such as hair and eyes.

#### 2.1.2. Facial Recognition and Normalization

In this study, the Dlib library was utilized to detect facial landmarks. Dlib is a C++ toolkit containing computer vision and machine learning algorithms that are used to identify the geometric structure of faces in digital images and to align faces through translation, rotation, and the scaling of images. It is widely applied in various domains. Research [11] shows that Dlib facial detection is based on Histograms of Oriented Gradients (HOGs). The HOG feature extraction technique was used to compute the statistical information about the direction of the local image gradients. Initially, the images were preprocessed and converted to grayscale, and then the gradients were calculated. Gamma correction techniques were applied to adjust the image contrast, thus reducing the impact of shadows and lighting on the image.

**Figure 1:**
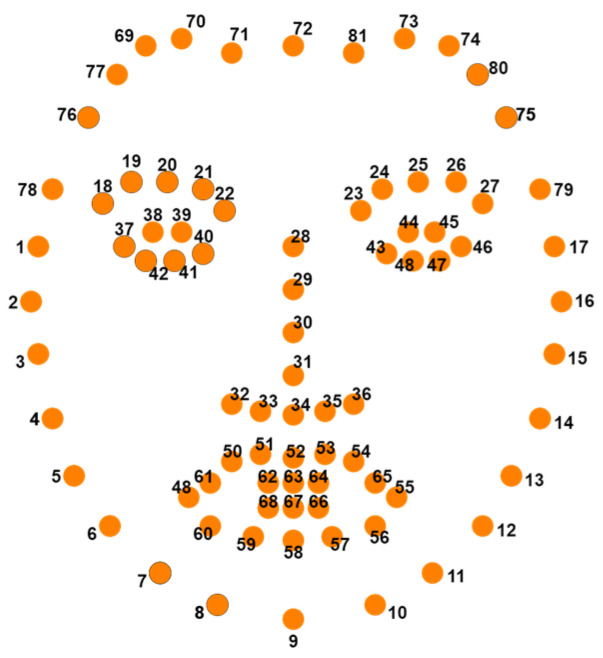
Dlib facial 81 landmark points diagram.

It is expected that the faces in a dataset will be focused on, and computer training datasets can locate facial landmarks through facial alignment and angle. There are primarily 68 (x, y) coordinates for facial landmarks; these map facial points distributed across the chin (1–17), right eyebrow (18–22), left eyebrow (23–27), nose (28–36), right eye (37–42), left eye (43–48), and mouth (49–68). This paper introduces an additional 13 points on the forehead (69–81) to improve accuracy in the facial recognition, as shown in Figure 1.

Normalization is the process of organizing data within a database. Its three main objectives are standardizing data scales, preventing numerical overflow, and enhancing algorithm stability. In this study, normalization involves computing the average value of each color channel in the ROI for every image frame. This step ensures signal normalization, thus facilitating stable computations in subsequent algorithms.

### 2.2. Signal Processing Algorithm

#### 2.2.1. Blind Source Separation (BSS) and Independent Component Analysis (ICA)

BSS [12,13] is a technique used to isolate the original signals from a mixture without prior information about the sources or their mixing process. BSS is applied across various fields, including image processing, telecommunications, biomedical engineering, and speech recognition. This study used independent component analysis (ICA) for a semi-blind source separation, where the mixing Matrix A is known. This knowledge facilitates a quicker computation of the separating Matrix W, enabling the isolation of independent component signals. However, uncertainties in the signal order and amplitude of the separated components persist. In summary, the described methods and experimental procedures leverage webcam-based face detection, ROI extraction, normalization, and blind source separation to compute blood pressure values accurately and efficiently.

**Figure 2:**
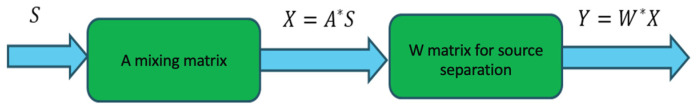
Independent component analysis architecture diagram.

ICA is a computational method based on statistical principles that aim to identify statistically independent and non-Gaussian factors or components within multidimensional data. Its classic problem is the cocktail party problem, which explores how humans can focus on specific conversations in a noisy cocktail party environment. The critical assumption of ICA is that the sources of signals are statistically independent, which holds in many cases of blind source signal separation. Even when this assumption is not fully met, ICA can still be utilized to achieve statistical independence among observed signals. ICA serves as a technique for blind source separation, where the aim is to find an appropriate separation Matrix W to compute independent-source Signal Y from a mixture of signals, as illustrated in Figure 2. A represents the unknown mixing matrix, while X and S represent the n-dimensional observed mixed and unknown source signals, respectively [14].

Waveform Processing: in this study, the extracted pulse wave undergoes ICA to eliminate the noise interference from the ambient light frequently encountered in RPPG measurements. Below are the detailed steps of the ICA process.

1. Input the measurement Signal X.
2. Centering: Subtract the mean from the input signal, $\hat{X}=X-E\left[X\right]$.
3. Whitening: $=V\hat{X}=VAS=\tilde{A}S$
4. Determine the number of independent components to be estimated, n.
5. Randomly select initial $W_{i}$, i = 1,…,n.
6. Simultaneously, perform Newton iteration updates on each $W_{i}$ and find the objective function $MaxJ\left(W^{T}Z\right)=E\left[G\left(W_{i}^{T}Z\right)\right]-E\left[G^{\prime }\left(W_{i}^{T}Z\right)\right]W_{i}$ [15].
7. Convergence criterion: Check if the user-defined iteration limit is reached. If so, output W and terminate. If not, return to Step 5 and continue until the convergence condition is met.
8. Obtain the independent component Signal Y, where $Y=W^{T}Z$.

**Figure 3:**
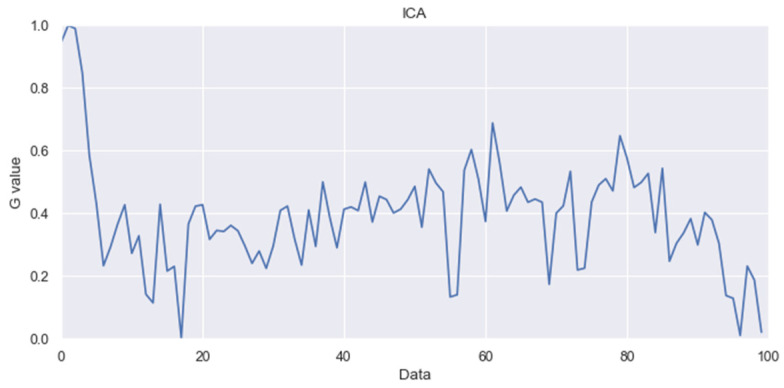
ICA waveform graph.

Pulse Wave Peak (Valley) Averages: blood pressure calculations are based on the processed waveforms after independent component analysis (as shown in Figure 3).

#### 2.2.2. PSO and NM-PSO

The NM method [16] compares various points during a search process. Through computational operations, each point moves toward the direction of the optimal point. If the path is correct, it can enclose the optimal solution, leading to rapid convergence. However, a drawback of this method arises when there are too many optimal solutions within the problem area, thus making it challenging to find the proper optimal solution and susceptibility to local optima [17]. The NM method first evaluates each point by substituting them into the evaluation function ff, thereby ranking them accordingly to find the best point Plow, the second-best point Psec hi, the worst point Phigh, and the centroid Pcent among them. Then, new points are generated through reflection, expansion, contraction, and shrinkage. After repeating these four steps, when the points are very close to the optimal solution, the convergence condition is reached and the algorithm terminates.

PSO was proposed by Kennedy and Eberhart in 1995 [18]. The concept of PSO originates from swarm behavior theory, i.e., it is inspired by observing the collective behavior of birds or fish, where individuals communicate information in a particular way, thus allowing the entire group to move toward a common direction. PSO is a method that mimics the social behaviors of individuals, imitating companions, and self-awareness to seek the optimal path. In the PSO algorithm, each group represents a possible solution to the optimization problem, where each bird or fish represents a particle. Each particle has the potential to become the best solution, and all particles have a fitness value representing the objective function value for the optimization problem, which is known as the fitness value. The PSO algorithm initializes with a group of random particles and iteratively finds the best solution for the group. In each evolution iteration, the particles update themselves by following the two best values: the individual best solution (Pbest) and the global best solution (Gbest). Pbest is the best solution found by the particle itself, while Gbest is the best solution found by the entire population. Each particle updates its velocity and position based on measurements referencing Pbest and Gbest. After generating new positions, measurements are taken multiple times until the optimal solution is produced [19].

(1) $$v_{id}\left(t+1\right)=w\times v_{id}\left(t\right)+rand_{1}\times c_{1}\times \left(Pbest-x_{id}\right)+rand_{2}\times c_{2}\times \left(Gbest-x_{id}\right),$$

(2) $$x_{id}\left(t+1\right)=x_{id}\left(t\right)+v_{id}\left(t+1\right).$$

- “i”: the i-th particle.
- “d”: the d-th dimension.
- “t”: the t-th measurement iteration.
- “w”: weight value.
- “$c_{1}$”, “$c_{2}$”: acceleration weight values.
- “rand1”, “rand2”: random numbers in the range [0, 1].
- “$v_{id}$(t)”: the velocity of the particle at the t-th measurement iteration.
- “$x_{id}\left(t\right)$”: the position of the particle at the t-th measurement iteration.
- “$v_{id}$(t + 1)”: the velocity of the particle at the (t + 1)-th measurement iteration.
- “$x_{id}$(t + 1)”: the position of the particle at the (t + 1)-th measurement iteration.
- “Pbest”: the best solution found by each particle individually over past measurements.
- “Gbest”: the best solution found by the entire particle swarm over past measurements.

The NM-PSO algorithm combines the PSO method with the NM method (which was proposed by Dr. Ho Yi-wei in 2004 [20]). The NM method has the advantage of a fast search speed, but it tends to become trapped in local optima. On the other hand, the PSO algorithm is less prone to local optima but may have a slower computation speed due to the need for more particles. The NM-PSO algorithm utilized in this study combines the strengths of the PSO and NM methods, resulting in faster computation speed and accurate identification of the optimal solution [21]. In the NM-PSO algorithm, 3N + 1 particles are initially generated to solve an N-dimensional problem. These particles are evaluated based on their positions, sorted, and divided into the N best particles, e.g., the (N + 1)th particle, and the 2N worst particles. Initially, the N best particles are preserved. Then, the NM method is applied to the N best particles and the (N + 1)th particle to obtain an updated (N + 1)th particle, which is then preserved. Subsequently, the preserved N best particles, the updated (N + 1)th particle, and the initial 2N worst particles are processed using the PSO algorithm. However, the updated results do not affect the preserved N best particles and the (N + 1)th particle; only the 2N worst particles are updated. This process is repeated until the stopping criteria are met.

**Figure 4:**
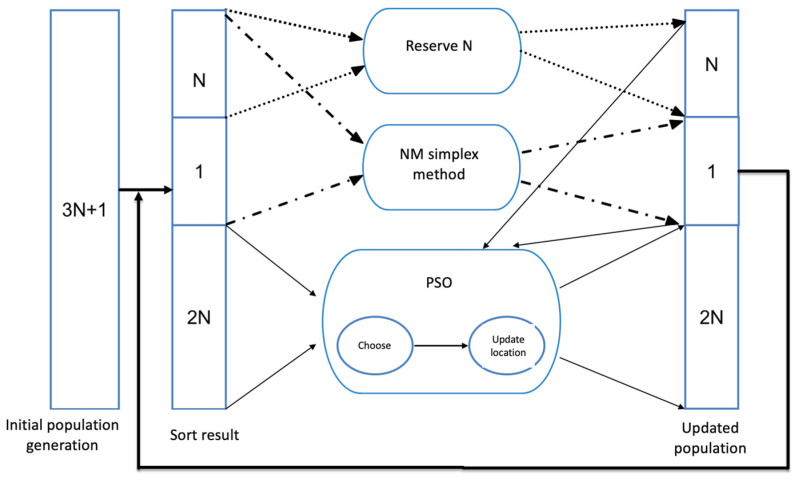
NM-PSO flowchart.

The detailed steps of the NM-PSO algorithm (as depicted in the flowchart in Figure 4) are as follows (the parameter settings are shown in Table 1):

1. Experiment Definition: Define the experimental parameters, select the evaluation function f*f*, set experimental parameters, and define the stopping criteria for the experiment.
2. Initial Generation: Generate a population of 3N + 1 candidate solutions if the dimension of the objective function is N.
3. Sorting: Evaluate the 3N + 1 particles using the evaluation function and sort them based on their performance, dividing them into N best particles, the (N + 1)th particle, and the 2N worst particles.
4. Preserve the N Best Solutions: Preserve the N best particles for updating the population and then wait to be updated along with other inferior solutions.
5. Update the (N + 1) Particle using the NM Algorithm: Apply the NM algorithm to update the N preserved best particles and the (N + 1)th Particle. The result will replace the (N + 1)th Particle and is preserved for updating the population.
6. Update using the PSO Algorithm: Update the population and the 2N worst particles using the PSO algorithm. However, the updated N best particles and the (N + 1)th Particle remain unchanged, and only the 2N worst particles are affected by the update. Store the updated 2N particles in the updated population.
7. Evaluate the Stopping Criteria: Check if the experiment’s stopping criteria are met. If satisfied, stop the experiment; otherwise, return to Step 3 to continue computation. Typically, the stopping criteria include the number of iterations or convergence of evaluation values.

### 2.3. Blood Pressure Measurement

#### 2.3.1. Principles of Non-Contact Blood Pressure Measurement

PPG is a non-invasive optical technique used to detect changes in blood volume in the micro-vessels of tissues. It relies on the principle of light absorption by human skin, which varies with the pulsatile blood volume. PPG measures the amount of blood passing through tissues with each heartbeat. The human skin consists of three layers: the epidermis with capillaries, the dermis with arterioles, and the subcutaneous tissue with arteries. Blood pressure is the lateral blood pressure within the blood vessels per unit area.

When a light beam passes through microvascular tissue, some of the light is absorbed by the body tissues due to reflection of or a penetration of the skin. The absorption of light can be described by the Beer–Lambert law, as shown in Equation (3):

(3) $$\varepsilon =\frac{Cl}{\log\left(\frac{I_{0}}{I}\right)},$$

where ε represents the absorption coefficient of light, C denotes the fluid concentration, l stands for the thickness of the absorbing medium, $I_{0}$ represents the radiation intensity, and I is the incident light intensity.

RPPG is a non-contact measurement technique based on the principles of PPG. It relies on a camera to measure the changes in red, green, and blue light reflected from the skin. The contrast between specular reflection and diffuse reflection is used, where specular reflection is pure light reflection from the skin, and diffuse reflection is the reflection left by the absorption and scattering of skin tissue, which changes with blood volume [22].

In the actual physiological system, there is blood viscosity, thickness of the aorta, and radial vibrations of arteries in the arterial system. Therefore, the heart is considered a periodic pressure pump based on Newton’s second law and the elasticity assumption. The Radial Resonance Theory (RRT) [23] was proposed in order that the arterial system is viewed as a transmission system for blood wave propagation and as a combination of various natural modes or inherent vibrations. RRT can provide a blood pressure calculation model and integrate the pulse wave signal with the body mass index to develop an extended linear model for blood pressure prediction.

Assuming that the arterial wall material properties follow Hooke’s law, the cross-sectional area of the tube wall remains circular under static pressure during the vibration process. It remains in the same plane, with only the diameter of the tube changing with time and axial position. In RRT, the radial arterial pressure is described as shown in Equation (4):

(4) $$p\left(z,t\right)=\overset{N}{\underset{k=0}{\sum }}\left[a_{k}\cos\left(\omega_{Hk}t\right)+b_{k}\sin\left(\omega_{Hk}t\right)\right]e^{-k\times \frac{z}{c}}$$

where $\omega_{Hk}$ represents angular frequency, z denotes the distance between the subject and the lens, c is the wave velocity, k indicates the number of waves, and $a_{k},b_{k}$ represents the amplitudes of waves. In the experimental signal, the waveforms of the zeroth and first instances were the most prominent, and higher-order waveforms were omitted. Therefore, it was simplified to Equation (5):

(5) $$p\left(t\right)=\left[a_{0}\cos\left(0\right)+b_{0}\sin\left(0\right)\right]e^{-0\times \frac{z}{c}}+\left[a_{1}cost+b_{0}sint\right]e^{-1\times \frac{z}{c}},\;=a_{0}+Q\cdot \cos\left(t\right)+R\cdot \sin\left(t\right)$$

where $a_{0}$ and $b_{0}$ represent the amplitudes of the zeroth-order waveform, while Q and R denote the amplitudes of the first-order waveform. The collected peaks $E_{peak}$ and valleys $E_{valley}$ form a time-series signal related to the pressure waves.

The peaks and valleys of the time-series signal were found to be highly correlated with the blood pressure. Firstly, the number of peaks and valleys was calculated, and then peaks and valleys caused by disturbances were removed. If assuming a sampling frequency of 20 FPS and a heart rate of 120 beats per minute (one beat every 0.5 s), there would be 10 data points per second. Since the interval between the peaks and valleys was 0.25 s, if the interval between any pair of peaks and valleys was to be less than 0.25 s, then that data point was discarded. Once the peak and valley values of the signal were obtained, their averages were calculated, as shown in Equations (6) and (7):

(6) $$E_{peak}=\frac{\Sigma_{n_{1}}H_{D}}{n_{1}},$$

(7) $$E_{valley}=\frac{\Sigma_{n_{2}}H_{L}}{n_{2}},$$

where $n_{1}$ and $n_{2}$ represent the number of peaks and valleys, respectively; $H_{D}$ denotes the sequence values of peaks; and $H_{L}$ denotes the sequence values of the valleys.

#### 2.3.2. Empirical Parameter Table for Blood Pressure Formula

**Figure 5:**
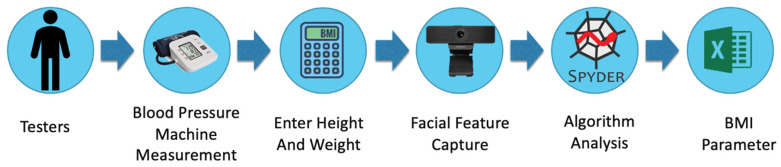
Flow chart for establishing the empirical parameters of the blood pressure formula.

In this study, we used EXCEL to establish an empirical parameter table for blood pressure formulas based on BMI values to facilitate the calculation of blood pressure values. Figure 5 illustrates the establishment of the empirical parameter table for blood pressure formulas. The empirical parameters for the blood pressure formulas in this system can be divided into the following five steps:

1. Human trials conducted in collaboration with a hospital in southern Taiwan (Ruan General Hospital Human Research Ethics Committee) were used as the measurement subjects for this study.
2. The test subjects first underwent blood pressure measurements using a monitor. Subsequently, their height and weight were entered into a computer to calculate BMI values. Facial features were then captured using a webcam, and the region of interest, specifically the forehead, was selected for extracting pulse wave signals.
3. The signals were normalized and adjusted, and independent component analysis was utilized to remove waveform artifacts.
4. The NM-PSO algorithm was employed in this study to calculate the empirical parameters in the formula.
5. BMI intervals separated these empirical parameters. After testing and adjustment, the empirical parameters within each interval were used as the formula parameters for that interval.

This study employed the blood pressure formula proposed in reference [24] to calculate the blood pressure values, as shown in Equation (8):

(8) $$f\left(x,BMI\right)=a_{0}+a_{1}\cdot x+a_{2}\cdot BMI+a_{3}\cdot x\cdot BMI,$$

where f(∙) is the approximation function, BMI represents the body metrics of the test subject, x denotes the average of all peaks (valleys), and $a_{0}$, $a_{1},\;a_{2},\;\mathrm{and}\;a_{3}$ are the empirical parameters.

To accelerate the computation speed of the blood pressure, this study derived a new formula by refining Equation (8), as shown in Equation (9), where the $a_{2}$ of Equation (9) is the $a_{2}$/$a_{3}$ of Equation (8):

(9) $$f\left(x,BMI\right)=a_{0}+a_{1}\cdot x+BMI*\left(1+a_{2}\cdot x\right),$$

where f (∙) is the approximation function, BMI represents the body metrics of the test subject, x denotes the average of all peaks (valleys), and $a_{0},\;a_{1},\mathrm{and}\;a_{2}$ are the empirical parameters.

Before computing the empirical parameters, the BMI values of the participants were calculated, and the blood pressure was measured using a blood pressure monitor. Simultaneously, the pulse wave signals, including peaks ($E_{peak}$) and valleys ($E_{valley}$) on the forehead, were extracted using a webcam attached to the computer. Subsequently, the BMI value, blood pressure measurements, and the $E_{peak}$ and the $E_{valley}$ of the participant were substituted into the fitness functions Equations (10) and (11) for SBP and DBP, respectively:

(10) $$SBP=a_{0}+a_{1}*E_{peak}+BMI*\left(1+a_{2}*E_{peak}\right),$$

(11) $$DBP=a_{0}+a_{1}*E_{valley}+BMI*\left(1+a_{2}*E_{valley}\right).$$

Then, the NM-PSO algorithm mentioned in this study was employed to determine the empirical parameters $a_{0},\;a_{1},\mathrm{and}\;a_{2}$.

Our experiments involved over 50 volunteer participants after dividing all the empirical parameters by BMI intervals. Therefore, this study used data expansion to increase the number of each interval to 20 groups. The empirical parameters within the interval were averaged and used as the formula for Equations (10) and (11).

### 2.4. Experimental Environment and Procedure

This study measured blood pressure using a webcam and computer processing. Daylight lamps provided the lighting with a brightness of 500 to 650 lumens. The experimental distance was approximately 65 cm. To ensure the accuracy of the experimental data, a contact blood pressure monitor was used for comparison during the measurements. The system environment is summarized in Table 2.

**Figure 6:**
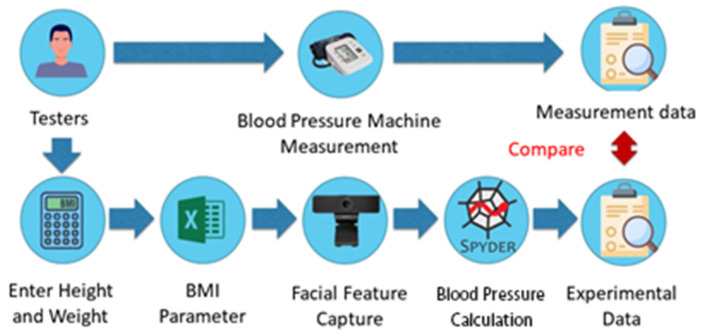
Non-contact blood pressure measurement system architecture diagram.

The system architecture of this research is illustrated in Figure 6. Initially, the test subjects input their height and weight into the computer to calculate their BMI values. Next, the corresponding blood pressure formula parameters for the BMI intervals were imported from an Excel spreadsheet. Subsequently, a webcam was used for face detection, and the pulse wave reflected from the forehead was captured within the region of interest. The blood pressure values were then calculated based on the extracted light wave. Additionally, blood pressure measurements were simultaneously taken using a blood pressure monitor to validate the accuracy of the calculated values.

**Figure 7:**
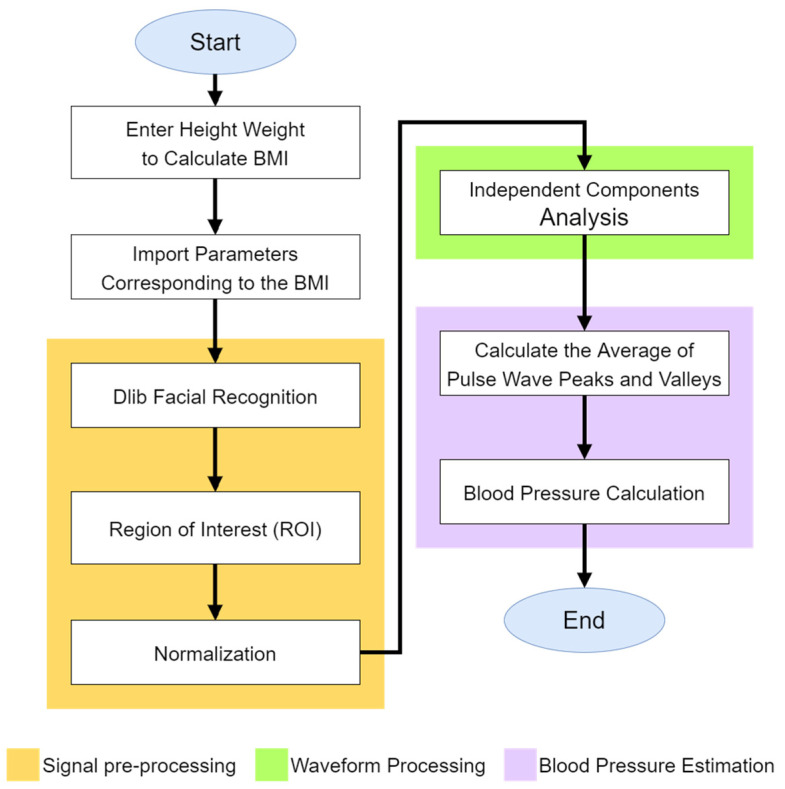
Non-contact blood pressure measurement process diagram.

**Figure 8:**
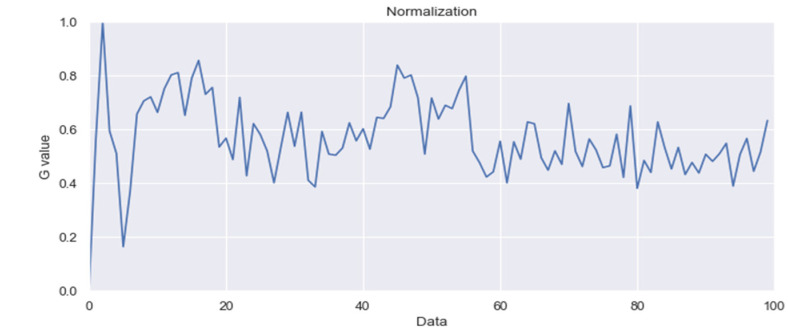
Normalized waveform analysis diagram.

The non-contact blood pressure measurement process in this study is illustrated in Figure 7. The system primarily comprised the following five steps:

1. Input Personal Information: The participant input their height and weight into the computer to calculate their BMI value, which is crucial for the blood pressure calculation in the system.
2. Facial Image Capture: Using a webcam, the system captured facial images of the participant. The Dlib library was used for detecting facial landmarks, specifically identifying 81 landmarks. The region of interest on the forehead (points 69–81) was selected for extracting the pulse wave signal.
3. Signal Processing: The captured signal underwent normalization to adjust the signal levels. Independent component analysis was then applied to remove waveform artifacts. Through Dlib’s detection of 81 facial landmarks in the facial images, the system then displayed the captured frames from the camera. Following this, the system extracted the region of interest on the forehead (points 69–81). Normalization was performed to mitigate the influence of head movements on the measurement accuracy, as depicted in Figure 8.

A single blood pressure measurement obtained 10 data points for the peaks (valleys). The average of these 10 data points was then calculated and used as the peak (valley) value for that measurement session to compute the blood pressure. The following were the steps undertaken for obtaining the peak (valley) values for the first data point. Initially, the system extracts all the values and lengths of the peaks (valleys) within one second of the pulse wave. According to this paper, totaling up all the peak (valley) values and dividing them by the number of data points yields the peak (valley) value for the first data point. Following the above two steps, ten continuous datasets were collected to calculate each set’s average peak (valley) value. The results were obtained by computing the required peak (valley) value for one blood pressure detection. The experimental values for a 10 s measurement are tabulated in Table 3.

In Table 3, “E_peak_len” is the length of the number of peak values, “E_valley_len” is the length of the number of valley values, “E_peak_sum” is the total of all peak values, “E_valley_sum” is the total of all valley values, “E_peak” is the peak value of the first dataset, and “E_valley” is the valley value of the first dataset.

4. Then, we used the NM-PSO algorithm and Equations (10) and (11) to find each BMI interval’s corresponding parameter values.
5. Substituted the peak and valley values obtained in the fourth step and those obtained by the NM-PSO algorithm for corresponding BMI intervals into Equations (10) and (11).

## 3. Experimental Results

In this experiment, 50 male and female volunteer participants were measured. The experiment focused on the facial region, with the testing distance between 65 cm and 100 cm. It was recommended that individuals with a BMI ranging from 17 to 29 should conduct measurements under an illuminance of 330 to 550 lux. The participants underwent non-contact blood pressure measurements and, simultaneously, the measurements were taken using blood pressure monitors certified by the Taiwan Ministry of Health and Welfare for comparison. Discrepancies between the measurements were examined to verify errors, and then, finally, the influence of the illuminance and measurement distance on the experimental results was explored.

### 3.1. Metrics

The experimental objective of this study was to establish a system that enables individuals to detect blood pressure anytime and anywhere. Therefore, the accuracy assessment of the system adopts MAPE, RMSE, and the Coefficient of Determination (R2) as evaluation indices, which are shown in Equations (12)–(14).

(12) $$MAPE=\frac{1}{N}\overset{N}{\underset{i=1}{\sum }}\frac{\left|\hat{y_{i}}-y_{i}\right|}{y_{i}}$$

(13) $$RMSE=\sqrt{\frac{1}{N}\overset{N}{\underset{i=1}{\sum }}{\left(y_{i}-\hat{y_{i}}\right)}^{2}}$$

(14) $$R^{2}=1-\frac{\sum_{i=1}^{n}{\left(y_{i}-{\hat{y}}_{i}\right)}^{2}}{\sum_{i=1}^{n}{\left(y_{i}-\bar{y}\right)}^{2}},$$

where *yi* represents the actual value of the ith sample, ${\hat{y}}_{i}$ represents the measured value of the ith sample, $\bar{y}$ represents the mean of all actual values, and *n* represents the total number of test samples.

### 3.2. Results

**Figure 9:**
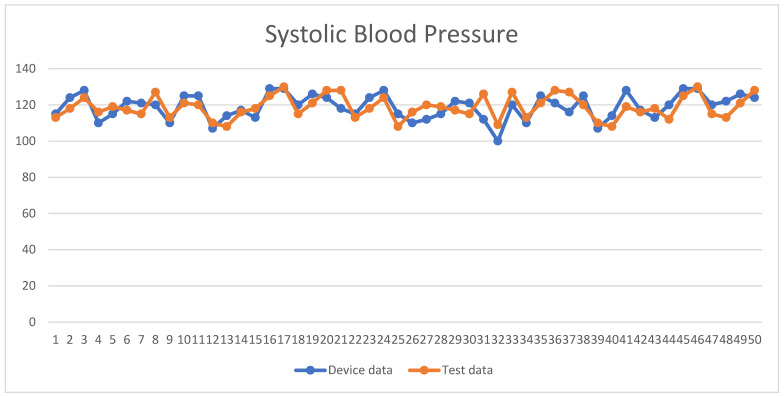
Line graph of the SBP data.

**Figure 10:**
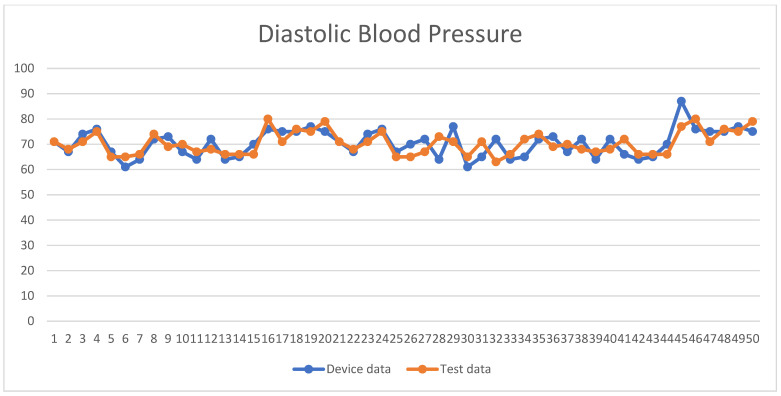
Line graph of the DBP data.

A total of 50 people were tested in this study, and the male-to-female ratio was approximately 2:1; the overall test results were not proportional to the male-to-female ratio, and the blood pressure values measured by the system were compared with those measured by a blood pressure monitor. The experimental and instrument-measured values, SBP and DBP, were recorded, as shown in Figure 9 and Figure 10, respectively. The *X*-axis represents the number of test subjects, and the *Y*-axis represents blood pressure values. The MAPE for the SBP in this study was 3.72%, the RMSE was 4.73 mmHg, the MAE was 4.67 mmHg, the STD was 3.04 mmHg, and the R2 was 0.50; for DBP, the MAPE was 3.9%, the RMSE was 3.12 mmHg, the MAE was 3.84 mmHg, the STD was 2.91 mmHg, and the R2 was 0.65, as shown in Table 4.

## 4. Discussion

This experiment successfully demonstrated the potential application of ICA combined with the NM-PSO algorithm in non-contact blood pressure monitoring. This combination demonstrates higher accuracy and reliability than a method using traditional PSO. This advantage lies in its ability to better handle individual differences and environmental interference in physiological signals. This method improves the accuracy of blood pressure estimation and demonstrates good adaptability and practicality.

### 4.1. Analysis of Relevant Factors

This study utilized the RPPG signal extracted from the reflection of light on the forehead. In this subsection, the experimental data under different conditions—i.e., measuring light intensities of 100 lux, 220 lux, and 550 lux, as well as distances of 30 cm, 65 cm, and 100 cm for light and distance factors—were explored as related issues.

#### 4.1.1. Light Intensity

The experimental method of this study relies on the principle of light reflection, where light intensity indirectly affects the experimental readings. The system tested three different light intensities of 100 lux, 220 lux, and 550 lux at a measurement distance of 65 cm. It was observed that higher accuracy was achieved under a 550-lux light intensity. A comparison of the errors is shown in Table 5.

#### 4.1.2. Distance

Regarding the experimental distance, if the region of interest for capturing is too far from the forehead, then the captured area will be smaller, leading to errors in light reflection values. This study fixed the light intensity at 550 lux, and the experiments were conducted at 30 cm, 65 cm, and 100 cm distances, respectively. At 30 cm and 100 cm distances, the lens may lose focus due to being too close or too far from the forehead. Therefore, this study found more accurate results at a measurement distance of 65 cm. A comparison of the errors is shown in Table 6.

### 4.2. Comparisons with Related Literature

According to the experimental results in the literature [25], the average absolute percentage error of systolic blood pressure is 8.07%, the root mean square error is 11.5 mmHg, the mean absolute percentage error for diastolic blood pressure is 6.54%, and the root mean square error is 6.24 mmHg. Measurements were taken at a distance of 150 cm for 30 s. Fang [7] used chromaticity signal processing and orthogonal skin plane methods to reduce noise and to extract image-based photoplethysmography signals. Frequency domain phase difference features were extracted from the signals obtained from the forehead and cheek regions using cross-correlation function and power spectral density methods. Subsequently, K-nearest neighbor (KNN) and linear regression models were used to model the blood pressure database based on frequency phase difference features to perform the blood pressure measurements, which were based on full-face images. The experimental results at a distance of 50 cm for 90 s showed that the average absolute percentage of the systolic blood pressure was 7.30%, the root mean square error was 10.12 mmHg, the mean absolute percentage error for diastolic blood pressure was 9.29%, and the root mean square error was 7.33 mmHg. In comparison, in our study, where the measurements were taken for only 10 s at a distance of 65 cm, the mean absolute percentage error for systolic blood pressure was 3.72%, the root mean square error was 4.73 mmHg, the mean absolute percentage error for diastolic blood pressure was 3.90%, and the root mean square error was 3.12 mmHg.

Several models with different structures were evaluated for accuracy in terms of SBP and DBP. According to the experimental results in [9,26,27,28], the comparative results indicate significant variations in performance across models, as reflected by the MAE and STD metrics. For systolic pressure predictions, the CNN-LSTM model demonstrated the lowest MAE (4.25 mmHg) and STD (5.91 mmHg). However, for diastolic pressure, the same model achieved the best results with the lowest MAE (2.18 mmHg) and STD (2.96 mmHg). Notably, the ICA-NMPSO model introduced in this work showed competitive performance for both systolic and diastolic pressure estimations, with MAE values of 4.67 mmHg and 3.84 mmHg, and STD values of 3.04 mmHg and 2.91 mmHg, respectively. This suggests that the ICA-NMPSO model holds advantages in accuracy and consistency, thus presenting a promising approach for clinical and remote monitoring applications, as presented in Table 7.

This study realized a low-cost system for non-contact blood pressure detection using a webcam, which was used to capture the RPPG physiological signal reflected from the forehead. However, issues such as the instability caused by insufficient light intensity during measurement and excessive movement could be addressed and improved by collecting more diverse data for different BMI ranges or by subdividing the BMI ranges further to enhance the system’s performance. We are adjusting the system parameters and algorithms based on other light intensities to mitigate the errors caused by insufficient light.

## 5. Conclusions

This paper provides an innovative artificial intelligence approach for estimating BP using the forehead RPPG signal. The proposed system has been designed to perform specific BP detection more efficiently, accurately, and rapidly than is the case in the published literature. In the future, exploring the integration of the proposed technology into smartphones, smart TVs, and other devices will enable widespread access to self-monitoring health statuses and promoting convenient and independent health management.

## Figures and Tables

**Table 1:** NM-PSO parameter configuration.

Parameters	Values
Reflectance parameters (α)	1
Expansion parameter (γ)	2
Contraction parameter (β)	0.5
Acceleration factors (c1, c2)	1.5
Inertia weight (W)	0.5
Number of particles	10
Number of iterations	100
Maximum velocity	120
Minimum velocity	0
Search maximum boundaries	120
Search minimum boundaries	0
Random numbers (rand1, rand2)	[0, 1]

**Table 2:** Experimental environment setup.

Parameters	Values
Operating system	Windows 10 (×64)
CPU	AMD Ryzen 5 5600 G
Memory	16 GB
Development environment	Tensorflow-Keras (Spyder4.2.0)
Programming language	Python3.7
Blood pressure monitor	OMRON HEM-8712
Webcam	E-books E-PCC072 (1080 p/30 fps)
Illuminance meter	Konica Minolta T-10

**Table 3:** Average peak (valley) values table.

TIME	E_peak_len	E_valley_len	E_peak_sum	E_valley_sum	E_peak	E_valley
1 s	30	30	5.43	4.16	0.18	0.13
2 s	29	28	13.82	10.40	0.47	0.37
3 s	30	29	7.67	4.81	0.25	0.16
4 s	31	31	16.48	12.89	0.53	0.41
5 s	31	31	17.05	13.81	0.55	0.44
6 s	30	30	7.33	4.05	0.24	0.13
7 s	29	28	22.60	17.28	0.77	0.61
8 s	32	32	17.90	7.41	0.52	0.22
9 s	35	35	26.93	20.50	0.76	0.58
10 s	31	32	22.53	16.54	0.72	0.51

**Table 4:** Experimental results for ICA-NM-PSO.

	SBP	DBP
R^2^	0.50	0.65
MAPE	3.72%	3.90%
RMSE(mmHg)	4.73	3.12
MAE (mmHg)	4.67	3.84
STD (mmHg)	3.04	2.91
Measurement Time	10 s	

**Table 5:** Comparison table of the light intensity measurements.

	SBP (mmHg)			DBP (mmHg)		
Light Intensity	Experimental Values	Machine Readings	Absolute Error	Experimental Values	Machine Readings	Absolute Error
110 lux	106	118	11.32%	59	65	10.16%
220 lux	110	120	9.09%	61	66	8.19%
550 lux	121	123	1.65%	70	68	2.85%

**Table 6:** Comparison table of the measurement distances.

	SBP (mmHg)			DBP (mmHg)		
Measurement Distance	Experimental Values	Machine Readings	Absolute Error	Experimental Values	Machine Readings	Absolute Error
30 cm	122	115	5.73%	60	66	9.09%
65 cm	112	114	1.78%	69	67	2.89%
100 cm	108	118	9.25%	55	66	20%

**Table 7:** Comparison of the BP estimation results of models with different structures.

Model	References	SBP (mmHg)			DBP (mmHg)		
		MAE	STD	R^2^	MAE	STD	R^2^
GRU	[26]	12.08	15.67	0.39	5.56	7.32	0.41
LSTM	[27]	6.90	5.43		6.50	5.12	
CNN-LSTM	[28]	4.25	5.91		2.18	2.96	
D1DC-LSTM	[9]	10.10	9.42		8.67	6.19	
Hybrid D1DCnet	[9]	8.36	6.22		5.69	3.97	
ICA-NMPSO	[This Work]	4.67	3.04	0.50	3.84	2.91	0.65

## Data Availability

The original contributions presented in the study are included in the article, further inquiries can be directed to the corresponding author.
